# Inhibition of human positive cofactor 4 radiosensitizes human esophageal squmaous cell carcinoma cells by suppressing XLF-mediated nonhomologous end joining

**DOI:** 10.1038/cddis.2014.416

**Published:** 2014-10-16

**Authors:** D Qian, B Zhang, X-L Zeng, J M Le Blanc, Y-H Guo, C Xue, C Jiang, H-H Wang, T-S Zhao, M-B Meng, L-J Zhao, J-H Hao, P Wang, D Xie, B Lu, Z-Y Yuan

**Affiliations:** 1 Department of Radiotherapy, Tianjin Medical University Cancer Institute and Hospital, National Clinical Research Center for Cancer, Key Laboratory of Cancer Prevention and Therapy, Tianjin, China; 2 Department of Lung Cancer, Tianjin Medical University Cancer Institute and Hospital, National Clinical Research Center for Cancer, Key Laboratory of Cancer Prevention and Therapy, Tianjin, China; 3 Department of Radiation Oncology, Bodine Cancer Center, Thomas Jefferson University School of Medicine, Philadelphia, PA, USA; 4 Department of Pancreatic Cancer, Tianjin Medical University Cancer Institute and Hospital, National Clinical Research Center for Cancer, Key Laboratory of Cancer Prevention and Therapy, Tianjin, China; 5 State Key Laboratory of Oncology in South China, Cancer Center, Sun Yat-Sen University, Guangzhou, China

## Abstract

Radiotherapy has the widest application to esophageal squamous cell carcinoma (ESCC) patients. Factors associated with DNA damage repair have been shown to function in cell radiosensitivity. Human positive cofactor 4 (PC4) has a role in nonhomologous end joining (NHEJ) and is involved in DNA damage repair. However, the clinical significance and biological role of PC4 in cancer progression and cancer cellular responses to chemoradiotherapy (CRT) remain largely unknown. The aim of the present study was to investigate the potential roles of PC4 in the radiosensitivity of ESCC. In this study, we showed that knockdown of PC4 substantially increased ESCC cell sensitivity to ionizing radiation (IR) both *in vitro* and *in vivo* and enhanced radiation-induced apoptosis and mitotic catastrophe (MC). Importantly, we demonstrated that silencing of PC4 suppressed NHEJ by downregulating the expression of XLF in ESCC cells, whereas reconstituting the expression of XLF protein in the PC4-knockdown ESCC cells restored NHEJ activity and radioresistance. Moreover, high expression of PC4 positively correlated with ESCC resistance to CRT and was an independent predictor for short disease-specific survival of ESCC patients in both of our cohorts. These findings suggest that PC4 protects ESCC cells from IR-induced death by enhancing the NHEJ-promoting activity of XLF and could be used as a novel radiosensitivity predictor and a promising therapeutic target for ESCCs.

Esophageal cancer is the eighth most common cancer and the sixth leading cause of cancer mortality worldwide. As the dominant type of esophageal cancer in China, esophageal squamous cell carcinoma (ESCC) still presents with a poor 5-year overall survival of <30%. Clinically, most ESCC patients present with locally advanced disease, for which definitive chemoradiotherapy (CRT) could be considered as the standard treatment.^1, 2, 3^ Radiotherapy has the widest application to ESCC patients and has a central role in the therapeutic strategy. However, the response of this disease to radiotherapy is variable and the treatment outcome is not sufficiently predicted by the existing diagnostic modalities.^4, 5, 6, 7^ Therefore, reliable molecular markers that predict response to radiotherapy have been highly desired to optimize therapeutic strategies and improve clinical outcomes.

DNA double-strand breaks (DSBs) are considered the most lethal DNA lesions produced by ionizing radiation (IR).^8^ Mammalian cells encompass two distinct pathways to repair DSBs: the nonhomologous end joining (NHEJ) pathway and the homologous recombination (HR) pathway.^9^ NHEJ acts during any phase of the cell cycle and is the main way to repair DSBs induced by IR.^10^ The NHEJ process is based on enzymes that capture the ends of the broken DNA, bring them together and finally repair the DNA damage. First, Ku70/Ku80 forms a heterodimer that binds the ends of the DSBs and recruits the core components (including DNA-PKcs, XRCC4/DNA ligase IV and XLF) to Ku-bound DNA ends. DNA ligase IV then repairs the breaks and forms a complex with XRCC4 and XLF.^11^ Previous studies have shown that the expression of many NHEJ-asociated proteins is deregulated in human cancers and helps to predict patient response to radiotherapy.^12, 13, 14^ Furthermore, the role of the NHEJ proteins in the resistance to IR has been reported both *in vitro* and *in vivo*.^15,16^ On this basis, the NHEJ pathway can be a promising target to improve the radiotherapy effect of human cancers.

The human positive cofactor 4 (PC4) and its yeast ortholog SUB1 (also named as coactivator p15) were initially identified *in vitro* as transcriptional coactivators.^17^ PC4 is located on chromosome 5p13^18^ and encodes a 127-amino acid protein that has an important role in various cellular processes including transcription, replication, DNA damage repair, chromatin organization and cell cycle progression.^17,19, 20, 21, 22^ PC4 has non-sequence-specific single-stranded and double-stranded DNA-binding abilities. It was reported that PC4 was recruited to DNA damage sites, induced by laser microirradiation, through its single-stranded DNA-binding capacity, which may be involved in the subsequent steps of DNA repair.^18^ Another study found that PC4 promoted ligase-mediated dsDNA ligation activity by end-joining assays with the XRCC4-ligase IV complex and enhanced the joining of noncomplementary DNA ends, suggesting its role as an activator in NHEJ and DSB repair activity.^23^ These data indicated that PC4 is implicated in the regulation of the DNA damage repair pathway, which is important for radiosensitivity of cancer cells, and may be a promising target to improve the prognosis and therapy for cancers. However, the role of PC4 in human tumorigenesis remains relatively unclear. Some studies have hypothesized that this nuclear protein is a tumor suppressor because PC4 prevented mutagenesis and killing by oxidative DNA damage.^18,24^ Other studies have shown that PC4 protein expression is upregulated in non-small cell lung cancer and could be an attractive new therapeutic target for the treatment of NSCLC.^25^ Up to now, however, the significance of PC4 expression in ESCC tissue and its effect on prognosis and therapy response have not been elucidated.

In this study, we reported, for the first time, that PC4 is upregulated in ESCC cells and clinical ESCC specimens. Our results showed that knockdown of PC4 increased the radiosensitivity of ESCC cells both *in vitro* and *in vivo* and suppressed cell NHEJ activity by downregulating expression of XLF. In addition, high expression of PC4 positively correlated with ESCC resistance to CRT and was a strong and independent predictor for poor disease-specifical survival of ESCC patients.

## Results

### The levels of PC4 modulate ESCC cell radiosensitivity *in vitro*

**Figure 1:**
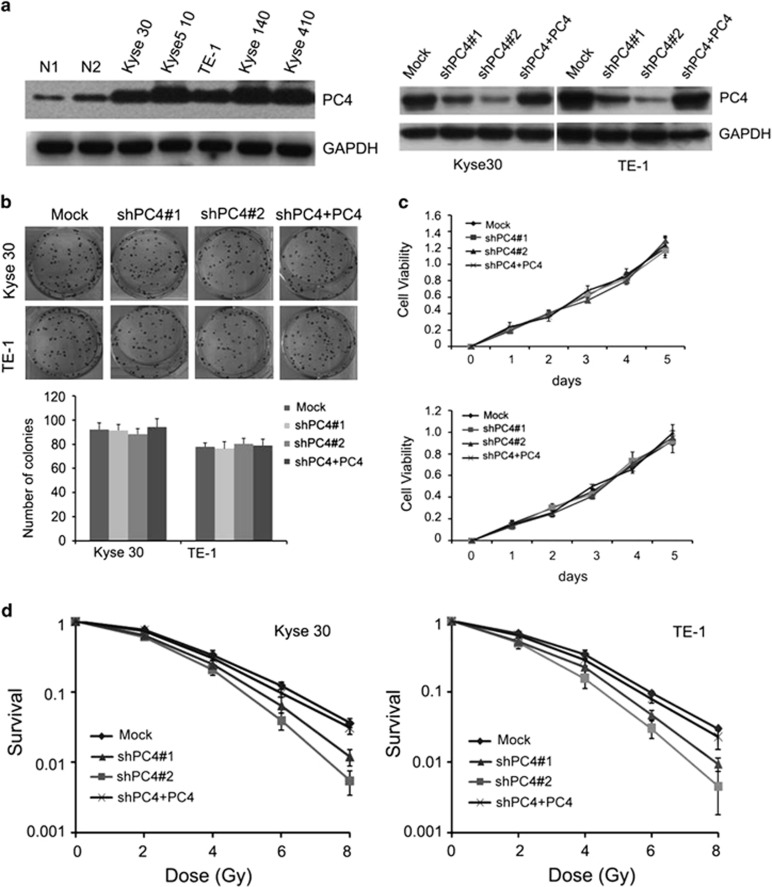
The levels of PC4 modulate ESCC cell radiosensitivity *in vitro*. (**a**, left) Western blot analysis showed that levels of PC4 in five ESCC cell lines (Kyse30, Kyse510, TE-1, Kyse140 and Kyse410) were higher than those in two normal esophageal epithelial cells (N1, N2). (**a**, right) Two shRNAs (shPC4#1 and shPC4#2) targeting *PC4* mRNA were introduced into two ESCC cell lines (Kyse30 and TE-1) for stable knockdown of PC4 through recombinant lentiviral infection. Then, pCDH-PC4 lentiviral particles were transduced into the above PC4-silenced ESCC cells (shPC4+PC4) to replenish PC4 expression. The levels of PC4 were examined by western blot. (**b**) The levels of PC4 have no effect on colony formation in Kyse30 and TE-1 cells. Surviving colonies (>50 cells/colony) were counted and are shown in a bar chart. (**c**) The levels of PC4 have no effect on the proliferation of Kyse30 and TE-1 cells. The cell viabilities were detected by MTT assay. (**d**) The responses of ESCC cells with different PC4 levels to ionizing RT were examined by clonogenic survival assay. Mock, non-silencing scramble RNA sequence control; shPC4, shRNA-targeting *PC4* mRNA. shPC4#2 had a better effect and was chosen for further study. Data represent the mean ±S.E. derived from three individual experiments with triplicate wells. Error bars, S.E.

Given the emerging view that PC4 is involved in DNA damage repair pathways and is aberrantly expressed in cancer, we were tempted to speculate that PC4 might have a potential role in the radiosensitivity of ESCC cells. In this study, we used western blot in five ESCC cell lines to initially examine protein levels of PC4. All five lines showed higher levels of endogenous PC4 than those of two normal esophageal epithelial cells (Figure 1a, left). Next, knockdown experiments were performed to explore whether PC4 influences the radiosensitivity of ESCC cells. We knocked down PC4 with lentiviral infection (shPC4#1 and shPC4#2) in Kyse30 and TE-1 cells, and shPC4#2 had a better silencing effect than shPC4#1 (Figure 1a, right). The colony formation and proliferation assay showed that knockdown of PC4 had no significant impact on the proliferative activity and colony formation capacity of untreated ESCC cells (Figures 1b and c). However, the survival capacity of PC4-knockdown ESCC cells was lower than that of the control cells after IR treatment (Figure 1d). shPC4#2 was chosen for further study because of its better radiosensitizing effect than that of shPC4#1. To make sure that the levels of PC4 modulate radiosensitivity of ESCC cells, we replenished the levels of PC4 in PC4-silenced Kyse30 and TE-1 cells by infection with recombinant lentivirus encoding a PC4 construct resistant to used short hairpin RNA (shRNA; shPC4#2) as described in Materials and Methods (Figure 1a, right). The results showed that after ectopic overexpression of PC4 in both PC4-silenced cells, the survival capacity of the cells under IR treatment was substantially enhanced (Figure 1d). These results suggested that increased expression of PC4 confers IR resistance to ESCC cells.

### Inhibiting expression of PC4 promotes IR-induced apoptosis and mitotic catastrophe

**Figure 2:**
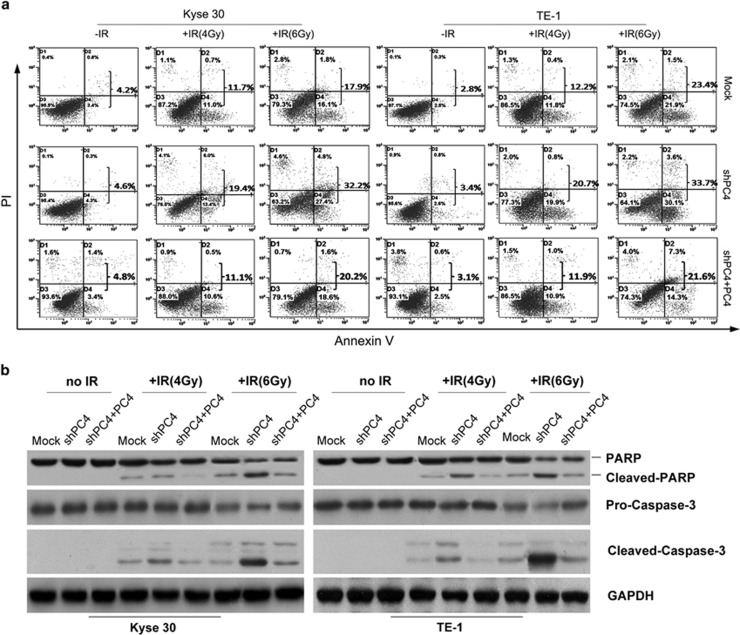
Silencing of PC4 promotes IR-induced apoptosis. (**a**) Silencing of PC4 increased the percentage of apoptotic cells in both Kyse30 and TE-1 cells after exposure to 4 and 6 Gy dose of IR. The IR-induced cell apoptotic death events were monitored by Annexin V/propidium iodide staining and flow cytometry assays. (**b**) Silencing of PC4 increased the levels of cleaved PARP and cleaved caspase-3 in both Kyse30 and TE-1 cells, which were exposed to 4 and 6 Gy dose of ionizing RT. Cleaved PARP and cleaved caspase-3 levels were determined by western blot

**Figure 3:**
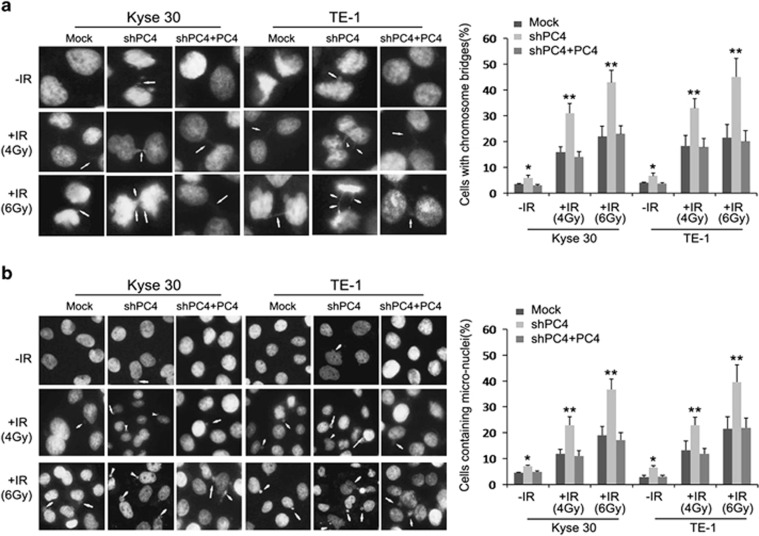
Silencing of PC4 promotes IR-induced MC in ESCC cells. Silencing of PC4 increased anaphase chromatid bridges (**a**) and micronuclei (**b**) of both Kyse30 and TE-1 cells, which were exposed to 4 and 6 Gy dose of ionizing RT. After being cultured in a normal medium for 24 h, cells were stained with DAPI and examined for chromosomes in mitosis (**a**) and nuclear morphology in interphase (**b**). Images were captured using a Nikon TE2000-U (Nikon Instruments Inc., Tokyo, Japan) inverted fluorescent microscope with a UV filter and processed by NIS-Elements software ( × 40 magnification; Nikon Instruments Inc.). Arrows indicate chromosome bridges in anaphase (**a**) and micronuclei in interphase (**b**). The percentage of cells with chromosome bridges (**a**) and cells containing micronuclei (**b**) were present at the bottom. An average of 400 cells in interphase and 200 cells in mitosis from three independent experiments were counted. The data represent mean values with S.E. (*P<0.05; **P<0.01, *P*-value was according to Student's *t*-test)

To test whether the PC4 knockdown-induced hypersensitivity to IR was due to activation of apoptotic death, we examined the effects of PC4 knockdown on apoptosis in Kyse30 and TE-1 cells using flow cytometry. Our result showed that silencing PC4 did not appear to induce cellular apoptosis in both cell lines. However, it significantly increased the proportion of apoptotic cells when cells were treated with IR (Figure 2a). Furthermore, western blot analysis suggested a significant increase in cleaved PARP and cleaved caspase-3 in PC4-silencing cells when cells were treated with IR (Figure 2b). Mitotic catastrophe (MC) has also been considered as another principal form of cell death induced by radiation.^26^ In this study, we therefore analyzed whether or not silencing PC4 affected ESCC cell response to IR-induced MC. Kyse30 and TE-1 cells were irradiated with 4 and 6 Gy irradiation dose of X-ray. After being cultured in a normal medium for 24 h, cells were stained by DAPI. Compared with both control cells, the incidences of anaphase chromatid bridges and micronuclei phenotype, two hallmarks of MC, were remarkably increased in PC4-knockdown Kyse30 and TE-1 cells treated with IR (Figures 3a and b). On the other hand, after replenishment of PC4 in both PC4-silenced Kyse30 and TE-1 cells, the altered apoptosis, cleaved PARP, cleaved caspase-3, anaphase chromatid bridge and micronuclei phenotypes were all recovered (Figures 2 and 3). These results, taken together, suggested that increased expression of PC4 confers on ESCC cells' resistance to IR-induced killing effects by avoiding apoptosis and MC.

### Silencing of PC4 increases ESCC-killing effect of IR *in vivo*

**Figure 4:**
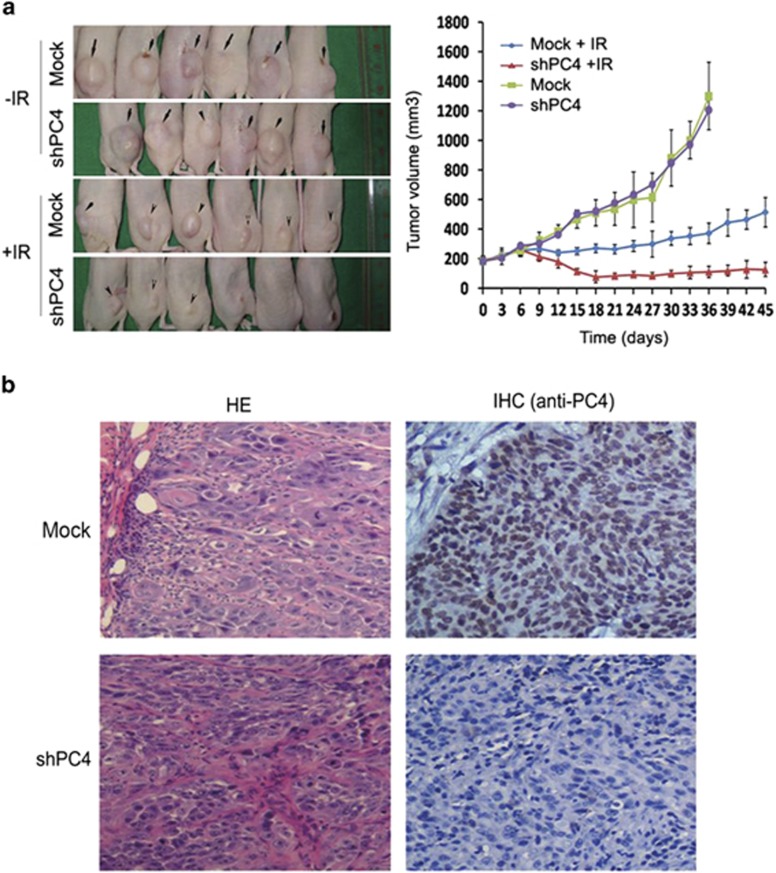
Inhibition of PC4 enhances the therapeutic effect of IR on ESCC cell xenografts. TE-1-shPC4 and control TE-1 cells (3 × 10^6^) were injected into the lower limb of female athymic nude mice. When the volume of a transplanted tumor reached 180 mm^3^, mice in the treatment groups were treated with a total 6 Gy dose of RT. Only the tumor, surrounding skin and subcutaneous tissues were exposed. (**a**) Tumor volume of xenografts was measured with calipers every 3 days for a total of 36–45 days. PC4 knockdown did not affect the growth of the tumors in the non-treatment groups (Mock and shPC4), and the mean tumor volume in the shPC4 and Mock groups was 1205.3±229.1 and 1298.6±289.8 mm^3^, respectively (*n*=6, *P*=0.53, Student's *t*-test). After a 6 Gy dose of IR treatment (groups of Mock+IR and shPC4#2+IR), the mean tumor volume in the shRNA group was 125.8±48.2 mm^3^, which was significantly smaller than that of 512.6±99.23 mm^3^ in the Mock group (*n*=6, *P*=0.003, Student's *t*-test). The values represent mean tumor volume±S.E. (**b**) Representative images showed xenograft tumors in null mice from TE-1-Mock and TE-1-shPC4#2 cells. H&E and IHC stainings of PC4 were performed on sections of tumors excised from mice after 36 and 45 days of treatment

We subsequently investigated whether PC4 knockdown could affect ESCC cell response to IR *in vivo*. TE-1-shPC4 and control TE-1 cells were inoculated into female athymic nude mice. When tumors reached a size of at least 180 mm^3^, the xenograft mice were treated with IR. Our results showed that knockdown of PC4 did not affect tumor growth of the untreated group. However, tumors developed more slowly in mice bearing the TE-1-shPC4 xenograft than in control mice after IR treatment (Figure 4). These results confirmed that PC4 protected ESCC cells from IR-induced death.

### Depletion of PC4 attenuates NHEJ though downregulating XLF expression in ESCC cells

**Figure 5:**
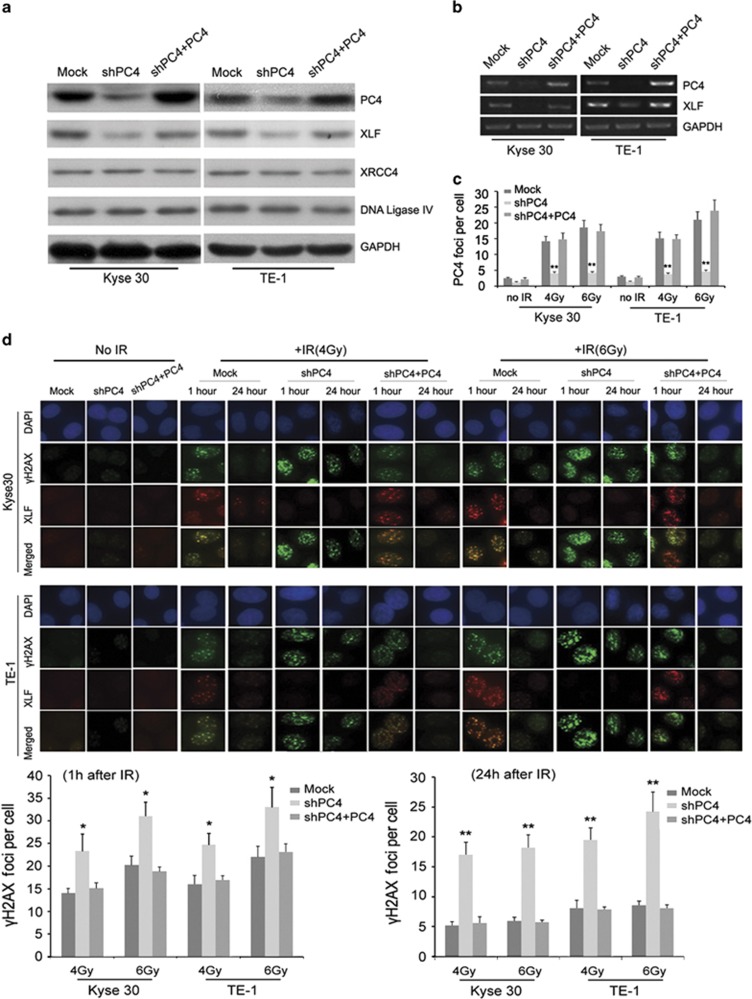
Silencing of PC4 inhibits the recruitment of XLF to DSB repair foci. (**a**) Inhibition of PC4 downregulates the protein expression of XLF. However, PC4 knockdown does not alter expression of XRCC4 and DNA ligase IV. GAPDH was used as a loading control. (**b**) Silencing of PC4 downregulates the mRNA expression of XLF. GAPDH was used as a loading control. (**c**) Silencing of PC4 inhibits IR-induced PC4 foci formation. The graph showed average numbers of PC4 foci per cell. Cells were subjected to IR (4 and 6 Gy) and 1 h later, fixed for staining with antibodies to PC4 and Immunofluorescence. (**d**, upper panel) Silencing of PC4 reduces the recruitment of XLF to IR-induced DSB repair foci. Cells were subjected to IR (4 and 6 Gy) and 1 or 24 h later, fixed for immunofluorescence. Shown is staining with antibodies to *γ*H2AX (green) and XLF (red). *γ*H2AX foci are used as a measure of DSB. Merged spots (yellow) show colocalization of XLF and *γ*H2AX foci at DSBs. (**d**, lower panel) Quantification of average numbers of IR-induced *γ*H2AX foci per cell (1 or 24 h after IR). Data were quantified by multiple counts and plotted. A total of 100 cells from three independent experiments were counted. The data represent mean values with S.E. (*P<0.05; **P<0.01, *P*-value was according to Student's *t*-test)

**Figure 6:**
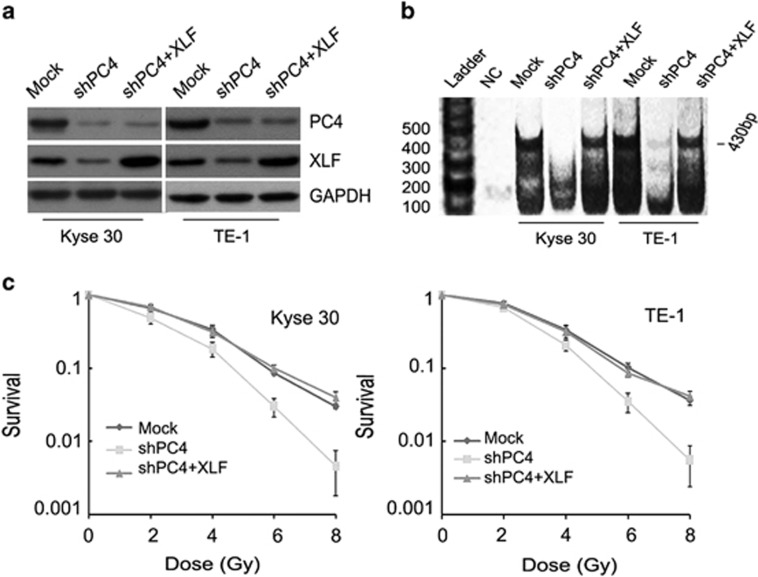
Silencing of PC4 attenuates NHEJ through depleting XLF expression in ESCC cells. (**a**) pCDH-XLF lentiviral particles were transduced into the above PC4-silenced ESCC cells (shPC4+XLF) to replenish XLF expression. The levels of PC4 and XLF were examined by western blot. (**b**) Inhibition of PC4 suppressed NHEJ in both Kyse30 and TE-1 cells, whereas replenishment of XLF in PC4-knockdown cells rescued cells' NHEJ activity. NHEJ assays were performed as described in Materials and Methods. The presence of 430 bp amplicons suggested that the NHEJ reaction had taken place. (**c**) The responses of ESCC cells to ionizing RT were examined by clonogenic survival assays. Cell resistance to IR was recovered in XLF-replenishment cells in which PC4 had been depleted. Data represent the mean±S.E. derived from three individual experiments with triplicate wells. Error bars, S.E.

NHEJ is a major pathway for the repair of IR-induced DNA DSBs in mammalian cells. PC4 helps in bringing the damaged DNA ends in close proximity for efficient ligation and thereby enhances NHEJ.^23^ This function of PC4 is similar to the final step of NHEJ by which the broken DNA ends are ligated through the collaboration of XLF, XRCC4 and DNA ligase IV.^27^ Therefore, we explored the possible effects of PC4 on this step of NHEJ. Inhibition of PC4 expression did not affect the expressions of XRCC4 and DNA ligase IV; however, as measured by both protein and mRNA levels, we showed that XLF was downregulated by PC4 knockdown in Kyse30 and TE-1 cells (Figures 5a and b). IR-induced foci formation of PC4 was also suppressed by inhibition of PC4 in both Kyse30 and TE-1 cells (Figure 5c). Furthermore, we asked whether downregulation of PC4 influences the foci formation and recruitment of XLF to the IR-induced DNA damage foci. XLF foci formation and colocalization with *γ*H2AX occurred as early as 1 h in Kyse30 and TE-1 cells treated with 4 and 6 Gy IR. Under the same condition, XLF foci formation decreased in Kyse30 and TE-1 cells whose PC4 expression was inhibited (Figure 5d, upper panel). On the contrary, after 1 or 24 h of IR treatment, the unrepaired DNA damage detected by *γ*H2AX in PC4-knockdown ESCC cells was significantly greater than that in the control cells after both 1 and 24 h of IR treatment (Figure 5d, lower panel). To further evaluate the effect of PC4 knockdown on NHEJ of ESCC cells, *in vitro* NHEJ assays were performed. Our result showed that silencing of PC4 (Figure 6a) indeed attenuated rejoining of noncomplementary DNA ends, as detected by the presence of 430 bp amplicons (Figure 6b). Interestingly, when XLF was overexpressed in PC4-knockdown ESCC cells by infecting with XLF lentivirus particles (Figure 6a), the inhibition of NHEJ and radiation-enhancing effect of PC4 deficiency was compromised (Figures 6b and c). Collectively, these data suggest that PC4 knockdown attenuates the NHEJ by downregulating XLF expression, which may be responsible for enhancing the radiosensitivity of ESCC cells.

### PC4 expression is correlated with treatment response of ESCC patients

The expression of PC4 was examined by immunohistochemistry (IHC) in 98 ESCCs of the learning cohort (from the Cancer Center of Sun Yat-Sen University), 46 ESCCs of the validation cohort (from Tianjin Medical University Cancer Institute and Hospital) and in 30 control normal esophageal mucosa samples (from the Cancer Center of Sun Yat-Sen University). High expression of PC4 was observed in 65.3% of ESCCs in the learning cohort and 58.7% in the validation cohort. However, only 23.3% of our normal esophageal mucosa samples showed high expression of PC4. Further, correlation analysis revealed that PC4 was a factor that showed a significant correlation with CRT response, in which high expression of PC4 was observed more frequently in the CRT-resistant group than in the CRT-sensitive group (*P*=0.001 and 0.005, respectively, Table 1). No significant association was found between PC4 expression with other clinicopathological features, such as patient age, gender and tumor grade (*P*>0.05, Table 1).

### High expression of PC4 predicts poor ESCC patient survival

**Figure 7:**
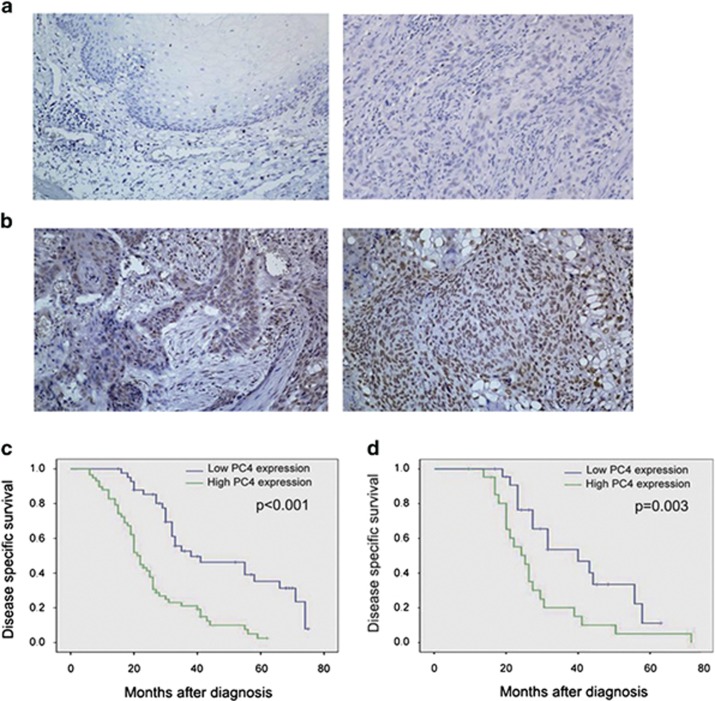
Immunohistochemical staining of PC4 in human esophageal tissues and its prognostic significance in ESCC patients. (**a**, left) Normal mucosa (case 8) in which <20% esophageal cells showed positive staining of PC4 in nuclei. (**a**, right) An ESCC (case 15 in the learning cohort) that exhibited low expression of PC4. (**b**, left) High expression of PC4 was examined in an ESCC (case 27 in the validation cohort), where >60% of carcinoma cells showed positive staining of PC4 in the nuclei. (**b**, right) High expression of PC4 was observed in another ESCC patient (case 67 in the learning cohort), where almost all carcinoma cells demonstrated positive staining of PC4. (**c** and **d**) High expression of PC4 was associated with poor prognosis of ESCC patients. Kaplan–Meier plots showed disease-specific survival curves of 98 ESCC patients in the learning cohort (**c**) and 46 ESCC patients in the validation cohort (**d**) treated with definitive CRT, according to PC4 expression levels in the primary tumor (*P*<0.01, log-rank test)

The mean observation period was 24.5 months (2.3–82.7 months) and 25.1 months (3.6–77.5 months) for learning and validation cohorts, respectively. During the course of this observation period, the number of cancer-related deaths was 70 and 41 for the learning and validation cohorts, respectively. In univariate analysis, high PC4 expression was evaluated to correlate closely with poor disease-specific survival (DSS) for both learning and validation cohorts (Figures 7c and d). Further, our multivariate analysis showed that PC4 expression and CRT response were independent predictors of patients' DSS in the learning cohort (*P*=0.012 and 0.003, respectively, Table 2), and the results were confirmed in our validation cohort (*P*=0.017 and 0.008, respectively, Table 2).

## Discussion

PC4 was originally identified as a transcriptional coactivator involved in various cellular processes such as transcription, replication and repair of DNA damage.^17^ To date, however, only a handful of reports have shown some contradictory results of PC4 in cancer progression and therapy response.^18,24,25^

Resistance of cells to chemotherapeutic agents and irradiation has been the main downfall of cancer treatments. Radiotherapy has been used extensively in treating locally advanced ESCCs, so we investigated whether PC4 expression could influence the radiotherapeutic sensitivity in ESCC cells. In the current study, our results clearly showed that PC4 knockdown could substantially increase ESCC cells' therapeutic response to IR *in vitro* and *in vivo*. To date, the potential mechanisms by which PC4 is involved in the radiosensitivity of human cancers have remained unclear. To our knowledge, apoptosis and MC, followed by serious or unrepaired DNA damage, are considered to be the major cell death mechanisms after IR in many solid tumors.^26,28, 29, 30^ Thus, we further examined the effects of PC4 on the IR-induced apoptosis and MC. Using flow cytometry, we found that inhibition of PC4 could enhance apoptotic cell death induced by IR. The incidences of anaphase chromatid bridges and micronuclei, two hallmarks of MC,^26,31,32^ also increased remarkably. These results strongly suggest that high expression of PC4 confers IR resistance to ESCC cells by preventing the cells from entering into apoptosis and MC.

NHEJ is the major pathway for the repair of IR-induced DNA DSBs in mammalian cells.^11,27^ Cells compromised for NHEJ exhibit genomic instability under stressful conditions and are, therefore, sensitive to radiation. Inhibition of NHEJ can be used as a means for making cancer cells hypersensitive to radiation. Many DNA-PKs inhibitors (such as IC86621 and NVP-BEZ236) showed dominant negative effects on both NHEJ and HR and are therefore excellent candidates for augmenting cancer radiotherapy.^33,34^ SCR7, another NHEJ inhibitor, could inhibit DNA ligase IV and improve efficacy of chemo- and radiotherapy.^35^

A previous study has shown that PC4 helps in bridging the DNA ends together and thereby brings them in close proximity for efficient ligation, where it enhances NHEJ and has an important role in DSB repair.^23^ However, Ku, the most important gene in the process of end joining, does not cooperate or compete with PC4.^23^ Further, potential mechanisms by which PC4 is involved in NHEJ have remained unclear. The ligation of the broken ends by DNA ligase IV is the final step in the repair of a DSB. In this process of NHEJ, XRCC4 and XLF form long, helical filaments that may serve to bridge or align DNA ends for ligation.^36, 37, 38, 39^ On this basis, we speculated that PC4 might take part in this final step of NHEJ. In this present study, we observed that after knockdown of PC4, XLF was downregulated, leading to a decrease in both XLF foci formation and the recruitment of this protein to DSBs under IR-induced DNA damage. This downregulation of XLF recruitment was coupled with an increase in the number of *γ*H2AX foci 24 h after IR, which is a hallmark of DNA damage. However, the foci formation and recruitment of XRCC4 and DNA ligase IV were not affected by PC4 knockdown (Supplementary Figure S1). A previous study has shown that downregulation of XLF in human cell lines leads to radiosensitivity and impaired NHEJ.^40^ Our result showed that both mRNA and protein expression of XLF was downregulated in our PC4-knockdown ESCC cells. As a transcriptional coactivator,^17^ PC4 may regulate the transcriptional activity of XLF and thus affect its expression. However, it is not clear how PC4 regulates XLF expression and further studies are obviously required. These findings, collectively, suggest that PC4 knockdown sensitizes ESCC cells to IR-induced DNA damage at least in part by negatively impacting the XLF-mediated NHEJ.

Interestingly, PC4 knockdown without IR treatment had no impact on the tumorigencity of ESCC cells *in vitro* or *in vivo*. This result was consistent with previous studies, which showed that PC4 is a non-essential gene that does not interfere with cell growth under normal physiological conditions.^17,18,41^ On this basis, developing potential PC4 inhibitors may provide better security than DNA-PKs inhibitors or other NHEJ inhibitors. Peng Y^25^ has reported that PC4 protein expression is upregulated in non-small cell lung cancer compared with their adjacent noncancerous counterparts. Our result also clearly showed that PC4 was frequently overexpressed in ESCC tissues. In the cohorts of our ESCC patients, high expression was found to correlate positively with ESCC resistance to CRT, and PC4 expression was a strong and independent predictor for short DSS of the disease. Thus, our reports suggested that the examination of PC4 expression could be used as an additional effective tool to predict the therapeutic response of ESCCs and optimize clinical decisions. Furthermore, we found that PC4 knockdown could also enhance ESCC cell sensitivity to cisplatin, which is the standard chemotherapeutic agent used in the CRT regimen of our clinical ESCC cohort (Supplementary Figure S2).

In summary, our reports describe, for the first time, that PC4 knockdown enhanced ESCC cell response to IR *in vitro* and *in vivo*. In addition, we provided evidence that PC4-mediated XLF expression, foci formation and recruitment to DSBs may promote NHEJ repair and account for the decrease of ESCC cell death by IR-induced apoptosis and/or MC. Moreover, our results provided a basis for the concept that high expression of PC4 may be a novel predictor of aggressive ESCC with CRT resistance and an independent prognostic factor for ESCC patients who are treated with definitive CRT. Thus, targeting PC4 may represent a new therapeutic strategy to improve the CRT effect and survival for ESCC patients.

## Materials and Methods

### Cell lines

Primary cultures of normal esophageal epithelial cells were established from fresh specimens of the adjacent noncancerous esophageal tissue according to previous studies.^42,43^ ESCC cell lines Kyse30, Kyse140, Kyse410, Kyse510 and TE-1 were obtained from Deutsche Sammlung von Mikroorganismen und Zellkulturen (DSMZ, Braunschweig, Germany), the German Resource Centre for Biological Material.^44^ Cells were cultured <3 months after resuscitation and were maintained in RPMI1640 supplemented with 10% fetal bovine serum and 1% penicillin–streptomycin at 37 °C in 5% CO_2_. Kyse30 and TE-1, mainly used in this study, were authenticated by short tandem repeat (STR) fingerprinting at China Center for Type Culture Collection (CCTCC, Wuhan University, Wuhan, China). A 320 kV X-ray machine (Precision X-Ray Inc., North Branford, CT, USA) delivered radiation at a dose rate of 2.3 Gy/min.

### Construction of the recombinant lentivrial vector

A plasmid containing the validated shRNAs targeting PC4 was cloned into the vector pLLU2G (kindly provided by professor Peng Xiang, Center for Cell Biology and Tissue Engineering, Sun Yet-Sen University). These vectors are derived from pLL3.7, contain separate GFP and shRNA expression elements and are required for lentiviral packging.^45^ The target sequences of PC4 for constructing lentiviral shRNA are 5′-GACAGGTGAGACTTCGAGA-3′ (shPC4#1) and 5′-TGAGGTACGTTAGTGTTCG-3′ (shPC4#2). For rescue experiments, a PC4 construct resistant to the used shRNA (shPC4#2; mutations underlined: TGCGATATGTATCGGTAAG, the mutations do not affect PC4 protein sequence) and an XLF construct were cloned into a pCDH cDNA expression lentivector (System Biosciences, Mountain View, CA, USA). The lentiviral expression construct and packaging plasmids' mix were then co-transfected into 293 cells to generate the recombinant lentivirus according to the manual.

### Clonogenic survival assay

Survival following radiation exposure was defined as the ability of the cells to maintain their clonogenic capacity and to form colonies. Briefly, after exposure to radiation, Kyse30 and TE-1ESCC cells were trypsinized, counted and seeded for colony formation in six-well plates with 50–5000 cells per well. After incubation intervals of 14–21 days, colonies were stained with crystal violet and manually counted. Colonies consisting of 50 cells or more were scored, and five replicate wells containing 10–150 colonies per well were counted for each treatment. Experiments were done in triplicate.

### Western blot assay

Cells were lysed in lysis buffer. A BCA kit (Pierce, Rockford, IL, USA) was used to determine protein concentrations. SDS-PAGE and western blot were done according to standard procedures. Proteins were detected with antibodies recognizing PC4, XLF, XRCC4 and DNA ligase IV (Abcam, Cambridge, MA, USA). GAPDH (Santa Cruz Biotechnology, Santa Cruz, CA, USA) was used as a loading control.

### Reverse transcription-PCR

The expression of PC4 and XLF mRNA was analyzed by a reverse transcriptase-PCR assay. The total RNA, which was extracted using TRIZOL (Invitrogen, Carlsbad, CA, USA), was used for cDNA synthesis with MMLV (Moloney murine leukemia virus) reverse transcriptase (Promega, Madison, WI, USA). cDNA was subjected to PCR with primers for PC4 (forward, 5′-GAGCCCTGTCATCTTCTA-3′ and reverse, 5′-TTCCTGGTTTCATTTCAC-3′), XLF (forward, 5′-TCCCAACATTTGATTCGTCCTC-3′ and reverse, 5′-GCCTTGATGCTTCTGTCCCAC-3′) and GAPDH (forward, 5′-GTTCGACAGTCAGCCGCATCT-3′ and reverse, 5′-CCTGCAAATGAGCCCCAGCCT-3′). Amplification consisted of 30 cycles of 30 s at 94 °C, 30 s at 56 °C and 60 s at 72 °C.

### MTT proliferation assay

Cell viability was measured with the use of a 3-(4, 5-dimethylthiazol-2-yl)-2, 5-diphenyl tetrazolium bromide (MTT) proliferation assay (Sigma, St. Louis, MO, USA). Briefly, cells were seeded in 96-well plates and cultured. Cell viability was examined following the standard procedures. Experiments were done in triplicate.

### Immunofluorescence

Cells grown on coverslips were fixed in 4% paraformaldehyde. Fixed cells were permeabilized with 1% Triton-100 and blocked with 10% normal goat serum. Cells were first incubated with primary antibody for 2 h at 37 °C in a humid chamber and then incubated with the secondary antibodies for 1 h. Immunofluorescence images were captured with FV10-ASW viewer software (Olympus, Tokyo, Japan). The primary antibodies that we used were *γ*H2AX (Cell Signaling, Danvers, MA, USA), XLF, XRCC4 and DNA ligase IV (Abcam).

### Annexin V-APC/propidium iodide flow cytometry apoptosis assay

Annexin V-APC and propidium iodide stains were used to determine the percentage of cells undergoing apoptosis. The apoptosis assay was conducted using the protocol supplied by the manufacturer (BD Biosciences, Bedford, MA, USA). Each sample was then subjected to analyses by flow cytometry (BD FACSCanto II Flow Cytometer, BD Biosciences).

### *In vivo* tumor growth assay

Female athymic nude mice (4- to 6-week old) were used for xenograft experiments. TE-1 shPC4 cells and the corresponding vector control TE-1 cells were injected subcutaneously on the lateral aspect of the rear leg. When tumors grew to the volume of 180 mm^3^, mice were randomized into four groups (six mice per group): Mock, shPC4, Mock+IR and shPC4+IR. In the groups of Mock+IR and shPC4+IR, a total radiation dose of 6 Gy (2 Gy per fraction every other day for 3 days) was delivered locally using a Pantak X-ray source (Precision X-Ray Inc.) to animals restrained in custom lead jig. Tumor diameters were measured with calipers every 3 days, and tumor volumes were calculated using the formula (width^2^ × length/2). All the procedures are in accordance with the guidelines of the laboratory animal ethics committee of Tianjin Medical University.

### NHEJ assay

An NHEJ assay was performed as described earlier.^23^ P21 cDNA was PCR amplified and digested with ApaI and PstI. The digested products (225 and 200 bp) with nonhomologous ends were incubated in NHEJ buffer for 30 min. Nuclear extract of our ESCC cells was then added to the mixed buffer and further incubated for another 30 min, followed by deproteination and precipitation of DNA. The precipitated DNA was used as template for PCR amplification using P21 gene-special primers. The presence of 430 bp products suggested that the NHEJ reaction had taken place. The PCR products were run on a 1% agarose gel and visualized after SYBR staining.

### Patients and tissue specimens

In this study, 98 ESCC patients who received definitive CRT at the Department of Radiotherapy at Sun Yat-Sen University Cancer Center between January 2002 and December 2008 were enrolled (learning cohort). As validation, we also studied 46 ESCC cases treated with definitive CRT in the same period from Tianjin Medical University Cancer Institute and Hospital (validation cohort). All patients received CRT with cisplatin-based chemotherapy and the same concomitant radiotherapy (daily dose of 1.8–2.0 Gy to a total dose of 60–70 Gy over 6–7 weeks). In addition, 30 biopsy samples of normal esophageal mucosa were used as controls. All the specimens were pretreated and recruited from paraffin blocks from the Department of Pathology of the two institutes. Tumor staging was carried out according to the 6th edition of the TNM classification of the International Union Against Cancer (UICC, 2002). The effect of CRT was evaluated clinically for primary lesions based on esophagography and computed tomography (CT) 4 weeks after treatment, according to the WHO criteria. The clinicopathological characteristics of the patients studied were summarized in Table 1. The study was approved by the medical ethics committee of the two institutes and with informed consent of the patients.

### Evaluation and follow-up

When patients completed the treatments, the effect of CRT was evaluated clinically for primary lesions based on esophagography and CT 4 weeks after treatment, according to the WHO criteria. Complete response, partial response, no change and progressive disease were achieved in 19 patients, 42 patients, 36 patients and 1 patient for the learning cohort, respectively, and in 10 patients, 19 patients, 16 patients and 1 patient for the validation cohort, respectively. Of the 115 patients who did not get CR, 31 cases received adjuvant chemotherapy and 4 cases received radical esophagectomy. The other patients did not receive any antitumor treatments until tumor progression. The patients were followed every 3 months for the first year, every 6 months for the next 2 years and annually thereafter. The diagnostic examinations consisted of esophagography, CT, chest X-ray, abdominal ultrasonography and bone scanning when necessary to detect recurrence and/or metastasis.

### Immunohistochemistry

IHC staining was performed as described earlier.^31^ The tissue slides were incubated with the rabbit polyclonal antibody against human PC4 (1 : 200, Abcam) overnight at 4 °C. Subsequently, the slides were sequentially incubated with biotinylated goat anti-rabbit immunoglobulin at a concentration of 1 : 100 for 30 min at 37 °C and then reacted with a streptavidin–peroxidase conjugate for 30 min at 37 °C and 3′-3′ diaminobenzidine as a chromogen substrate. The nucleus was counterstained using Meyer's hematoxylin. A negative control was obtained by replacing the primary antibody with a normal rabbit IgG. Positive expression of PC4 in ESCC and normal esophageal mucosa cells was primarily nuclear patterns (Figures 6a and b). Known immunostaining-positive slides were used as positive controls. Two independent observers, blinded to the clinicopathological information, performed scoring using a scoring system^31^ by which the percentage of nuclei staining positive for the PC4 protein, irrespective of staining intensity, was classified into two groups: low expression, in which fewer than 50% of cells were PC4 positive (Figure 6a), and high expression, in which at least 50% of the cells showed positive immunoreactivity of PC4 in the nuclei (Figure 6b). In this IHC study, a minimum of 500 epithelial cells were counted for each normal or tumor case.

### Statistical analysis

Statistical analysis was performed with the SPSS13.0 statistical software package (IBM, Armonk, NY, USA). Data derived from cell line experiments are presented as mean±S.E. and assessed by the two-tailed Student's *t*-test. The associations between PC4 expression and ESCC patients' clinicopathological features were assessed by the *χ*^2^-test. DSS was defined as the time from diagnosis to cancer-related death, analyzed with the Kaplan–Meier method and compared by the log-rank test. Multivariate survival analysis was performed on all parameters that were found to be significant on univariate analysis using the Cox regression model. *P*<0.05 was considered statistically significant.

## Figures and Tables

**Table 1:** Clinicopathological correlation of PC4 expression in ESCCs

**Variables**	**Learning cohort**			**Validation cohort**		
	**Cases**	**High expression (%)**	***P-*****value**^a^	**Cases**	**High expression (%)**	***P***-**value**^a^
*Age (years)*			0.754			0.845
⩽55^b^	54	36 (66.7)		25	15 (60)	
>55	44	28 (63.6)		21	12 (57.1)	
*Gender*			0.405			0.583
Male	82	55 (67.1)		38	23 (63.2)	
Female	16	9 (56.3)		8	4 (50)	
*WHO grade*			0.945			0.860
G1	24	15 (62.5)		11	7 (63.6)	
G2	50	33 (66)		20	12 (60)	
G3/4	24	16 (66.7)		15	8 (53.3)	
*Tumor size (cm)*			0.540			0.611
⩽6^c^	56	38 (67.9)		32	18 (56.2)	
>6	42	26 (61.9)		14	9 (64.2)	
*T status*			0.741			0.774
T2	12	9 (75.0)		12	6 (50)	
T3	35	22 (62.9)		18	11 (61.1)	
T4	51	33 (64.7)		16	10 (62.5)	
*N status*			0.615			0.428
N0	17	12 (70.5)		14	7 (50)	
N1	81	52 (64.2)		32	20 (62.5)	
*M status*			0.273			0.515
M0	59	36 (61.0)		24	13 (54.1)	
M1-lym	39	28 (71.8)		22	14 (68.1)	
*CRT response*			0.001			0.005
CR	19	6 (31.6)		10	2 (20)	
Not CR	79	58 (73.4)		36	25 (69.4)	

Abbreviations: CR, complete response; M, metastases; M1-lym, distant lymph node metastasis; N, node; T, tumor

a *χ*^2^-test

b Mean age

c Mean tumor size

**Table 2:** Multivariate Cox regression analysis for DSS in ESCC patients

**Factors**	**Learning cohort**			**Validation cohort**		
	**HR**	**95% CI**	***P*****-value**	**HR**	**95% CI**	***P-*****value**
PC4 expression	2.178	1.096–4.008	0.012	2.229	1.614–4.993	0.017
CRT response	3.271	1.485–7.207	0.003	3.958	1.386–7.667	0.008
N stage	1.233	0.586–2.595	0.581	1.663	0.562–3.893	0.329
M stage	1.595	0.946–2.691	0.080	1.331	0.667–2.433	0.039

Abbreviations: CI, confidence interval; DSS, disease-specific survival; HR, hazard ratio

## Abbreviations

ESCC: esophageal squamous cell carcinoma
PC4: human positive cofactor 4
NHEJ: nonhomologous end joining
CRT: chemoradiotherapy
MC: mitotic catastrophe
DSBs: DNA double-strand breaks
HR: homologous recombination
IR: ionizing radiation
DSS: disease-specific survival
