# Supplementary figures and images for: Inhibition of human positive cofactor 4 radiosensitizes human esophageal squmaous cell carcinoma cells by suppressing XLF-mediated nonhomologous end joining
Source: Cell Death Dis. 2014 Oct 16;5(10):e1461–. doi: 10.1038/cddis.2014.416 (PMC4649520; doi:10.1038/cddis.2014.416)

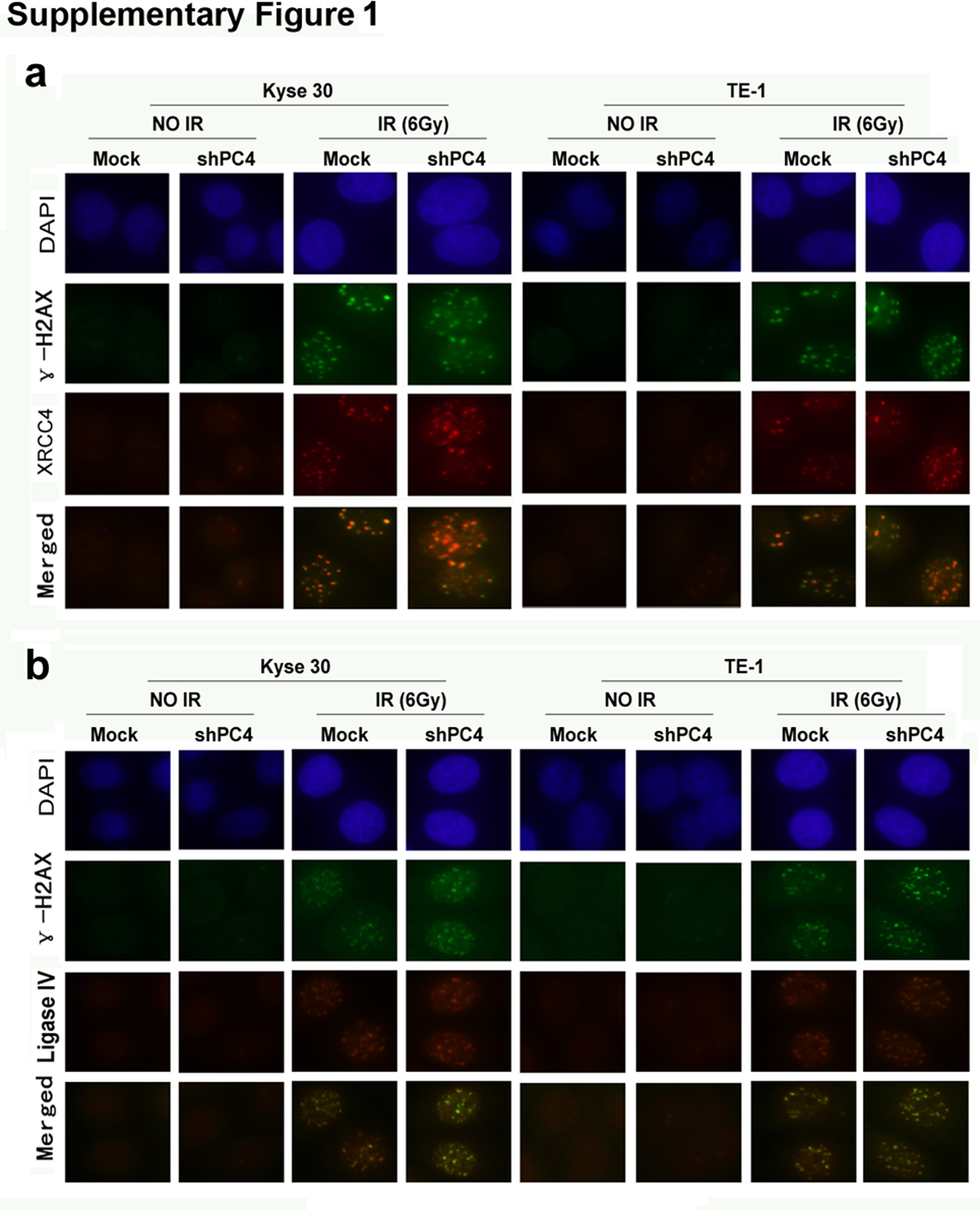

Supplement: Supplementary Figure S1 [file cddis2014416x1.tif]

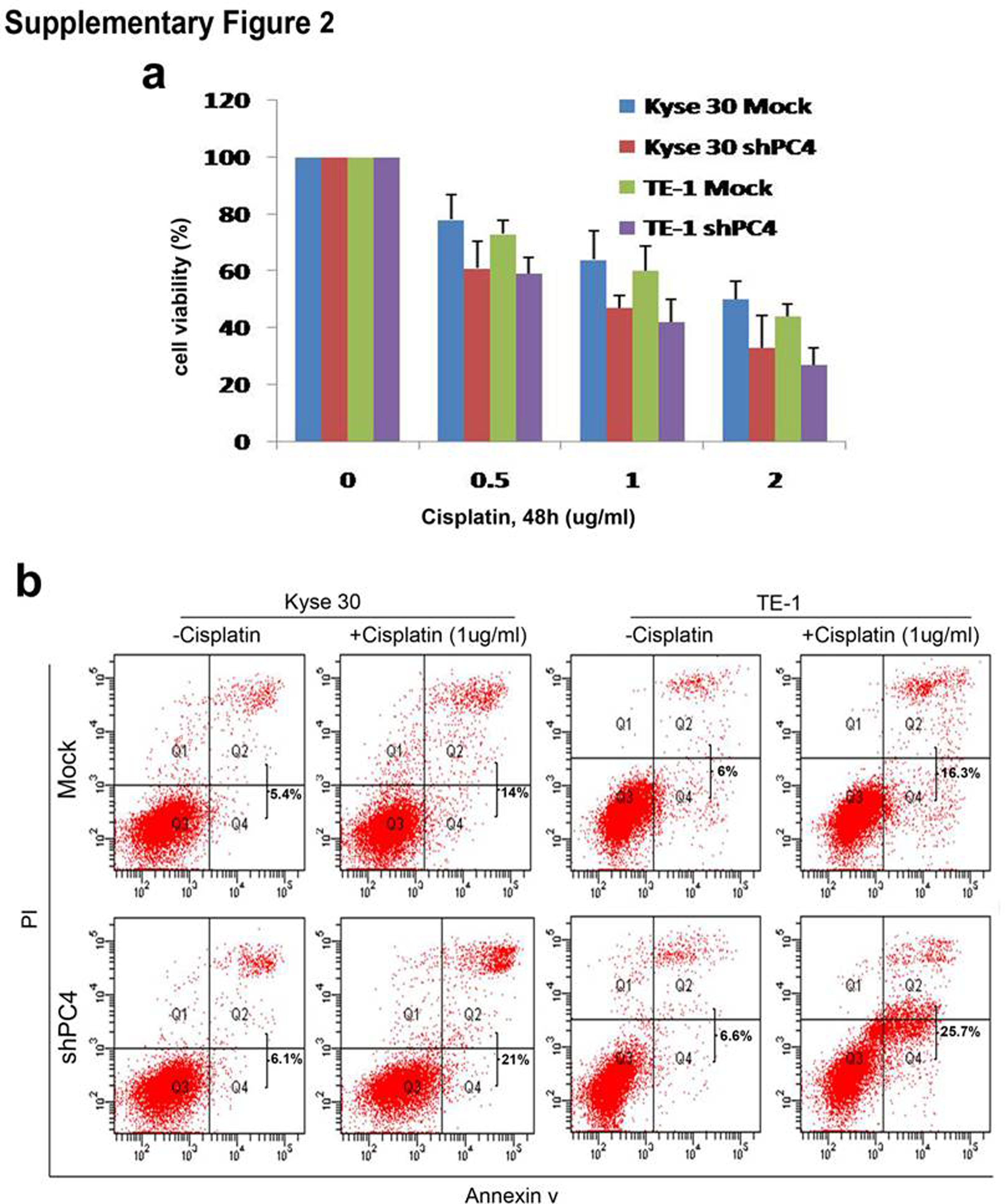

Supplement: Supplementary Figure S2 [file cddis2014416x2.tif]
