# Supplementary material for: Inhibition of human positive cofactor 4 radiosensitizes human esophageal squmaous cell carcinoma cells by suppressing XLF-mediated nonhomologous end joining
Source: Cell Death Dis. 2014 Oct 16;5(10):e1461–. doi: 10.1038/cddis.2014.416 (PMC4649520; doi:10.1038/cddis.2014.416)
Supplement: Supplementary Figure Legends [file cddis2014416x3.doc]

**Supplementary Figure legends**

**Supplementary Figure S1: Inhibition of PC4 have no affect on the recruitment of XRCC4 and DNA Ligase IV to DSB repair foci. a**, Silencing of PC4 does not alter the recruitment of XRCC4 to IR induced DSB repair foci. Cells were subjected to IR (6Gy), 1 hour later, fixed for Immunofluorescence. Shown is staining with antibodies to γH2AX and XRCC4. **b**, Silencing of PC4 does not alter the recruitment of DNA Ligase IV to IR induced DSB repair foci. Cells were subjected to IR (6Gy), 1 hour later, fixed for Immunofluorescence. Shown is staining with antibodies to γH2AX and DNA Ligase IV.

**Supplementary Figure S2: The impact of PC4 inhibition on ESCC cells chemosensitivities to cisplatin. a**, Kyse30-shPC4 and TE-1-shPC4 and corresponding control cells were treated with cisplatin for 48 h at the indicated concentration. The cell viabilities were detected by MTT assay. Data represent the mean ± SE derived from 3 individual experiments with triplicate wells. **b**, The same cells were treated with indicated doses of cisplatin (1ug/ml) for 48 h, and then the drugs-induced cell apoptotic events were monitored by Annexin V-APC/PI staining and flow cytometry assays (the rate of cell apoptosis=D2％+D4％). All data represent the mean ± SE derived from 3 individual experiments with triplicate wells.
